# Anticipating epidemic transitions with imperfect data

**DOI:** 10.1371/journal.pcbi.1006204

**Published:** 2018-06-08

**Authors:** Tobias S. Brett, Eamon B. O’Dea, Éric Marty, Paige B. Miller, Andrew W. Park, John M. Drake, Pejman Rohani

**Affiliations:** 1 Odum School of Ecology, University of Georgia, Athens, Georgia, United States of America; 2 Center for the Ecology of Infectious Diseases, University of Georgia, Athens, Georgia, United States of America; 3 Department of Infectious Diseases, University of Georgia, Athens, Georgia, United States of America; University of Chicago, UNITED STATES

## Abstract

Epidemic transitions are an important feature of infectious disease systems. As the transmissibility of a pathogen increases, the dynamics of disease spread shifts from limited stuttering chains of transmission to potentially large scale outbreaks. One proposed method to anticipate this transition are early-warning signals (EWS), summary statistics which undergo characteristic changes as the transition is approached. Although theoretically predicted, their mathematical basis does not take into account the nature of epidemiological data, which are typically aggregated into periodic case reports and subject to reporting error. The viability of EWS for epidemic transitions therefore remains uncertain. Here we demonstrate that most EWS can predict emergence even when calculated from imperfect data. We quantify performance using the area under the curve (AUC) statistic, a measure of how well an EWS distinguishes between numerical simulations of an emerging disease and one which is stationary. Values of the AUC statistic are compared across a range of different reporting scenarios. We find that different EWS respond to imperfect data differently. The mean, variance and first differenced variance all perform well unless reporting error is highly overdispersed. The autocorrelation, autocovariance and decay time perform well provided that the aggregation period of the data is larger than the serial interval and reporting error is not highly overdispersed. The coefficient of variation, skewness and kurtosis are found to be unreliable indicators of emergence. Overall, we find that seven of ten EWS considered perform well for most realistic reporting scenarios. We conclude that imperfect epidemiological data is not a barrier to using EWS for many potentially emerging diseases.

## Author summary

Anticipating disease emergence is a challenging problem, however the public health ramifications are clear. A proposed tool to help meet this challenge are early-warning signals (EWS), summary statistics which undergo characteristic changes before dynamical transitions. While previous theoretical studies are promising, and find that epidemic transitions are preceded by detectable trends in EWS, they do not consider the effects of imperfect data. To address this, we developed a simulation study which assesses how case aggregation and reporting error impact on 10 different EWS’s performance. Case report data were simulated by combining a stochastic SIR transmission model with a model of reporting error. Temporal trends in an EWS were used as a method of distinguishing between an emerging disease (*R*_0_ approaching 1) and a stationary disease (constant *R*_0_). We investigated the robustness of EWS to reporting process parameters, namely the aggregation period, reporting probability and overdispersion of reporting error. Seven of ten EWS perform well for realistic reporting scenarios, and are strong candidates for incorporation in disease emergence monitoring systems.

## Introduction

There are numerous causative factors linked with disease emergence, including pathogen evolution, ecological change and variation in host demography and behavior [1–5]. Combined, they can make each pathogen’s emergence seem idiosyncratic. In spite of this apparent particularity, there is a recent literature on the possibility of anticipating epidemic transitions using model-independent metrics [6–14]. Referred to as early-warning signals (EWS), these metrics are summary statistics (e.g. the variance and autocorrelation) which undergo characteristic changes as the transition is approached. In addition to infectious disease transmission, EWS have been investigated for transitions in a broad range of dynamical systems, including ecosystem collapse and climate change [15–21]. The motivation for EWS comes from the theories of dynamical systems and stochastic processes, in particular the slowing down that universally occurs in the vicinity of dynamical critical points [22–24]. Theoretical results for disease emergence are promising, and suggest that the transition from limited stuttering chains of transmission (*R*_0_ < 1) to sustained transmission and outbreaks (*R*_0_ > 1) is preceded by detectable EWS [8, 13, 14].

**Fig 1:**
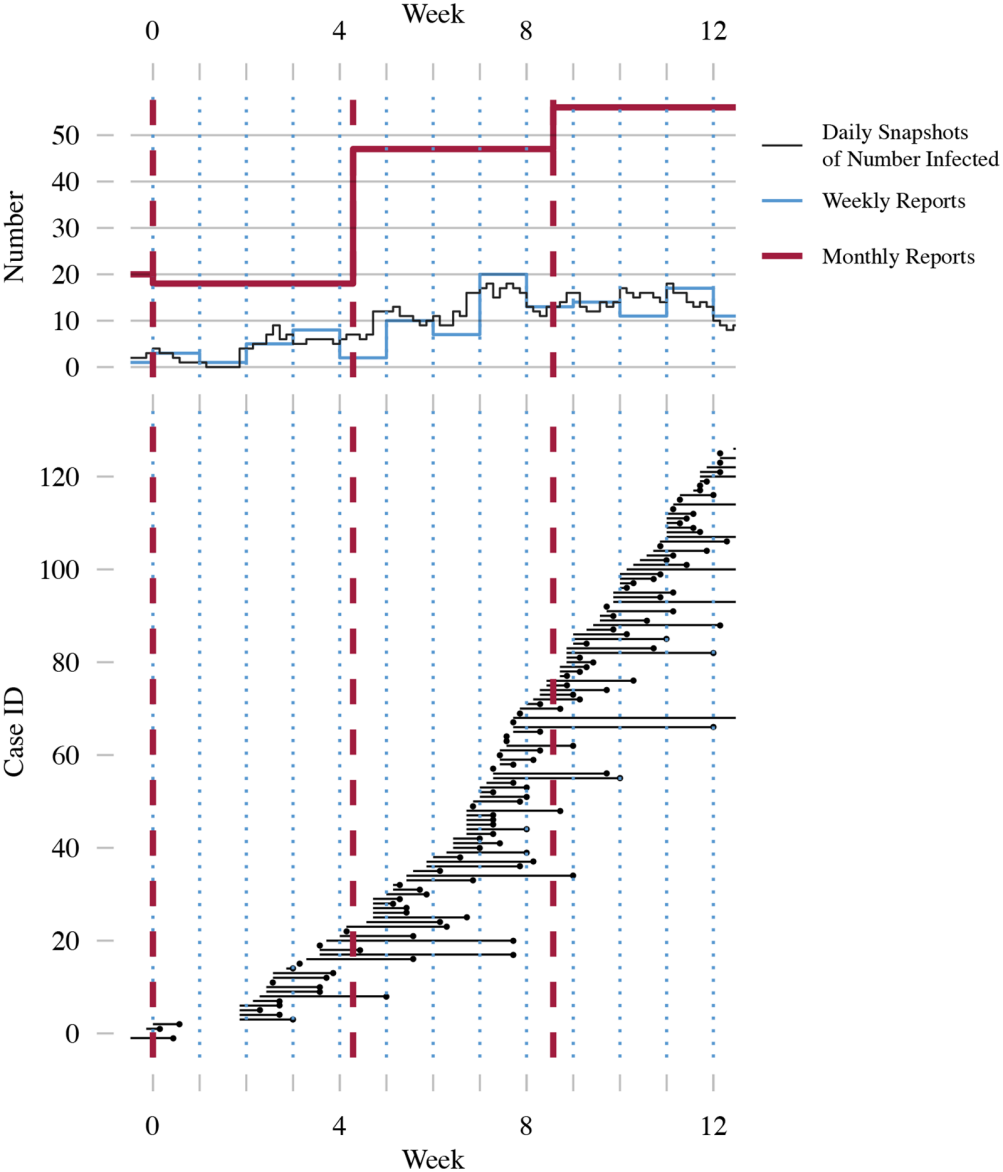
Demonstration of aggregation effects in epidemiological data. The bottom panel shows the progression of a simulated outbreak in a population, with cases ranked by their time of infection. Solid black lines indicate the duration of infectiousness, dots indicate time of recovery. The top panel shows three time series calculated from the simulated data: daily snapshots of the number of infected present in the population (black), weekly case reports (blue) and monthly case reports (red). For the purposes of this paper, the number of recovery events falling within an aggregation period serves as a proxy for the true number of cases in a case report. Aggregation periods are delimited by blue dots for weekly reports and red dashes for monthly reports. No reporting error is applied to the case reports shown in this figure. Transmission dynamics are modeled using the SIR model with birth and death with average population size *N*_0_ = 10^6^, importation rate *ζ* = 1 case per week, mean life expectancy of 70 years, and mean infectious period of 1 week. *R*_0_ increases linearly from 0 to 1 over 20 years. Simulations performed using the Gillespie algorithm [33].

**Fig 2:**
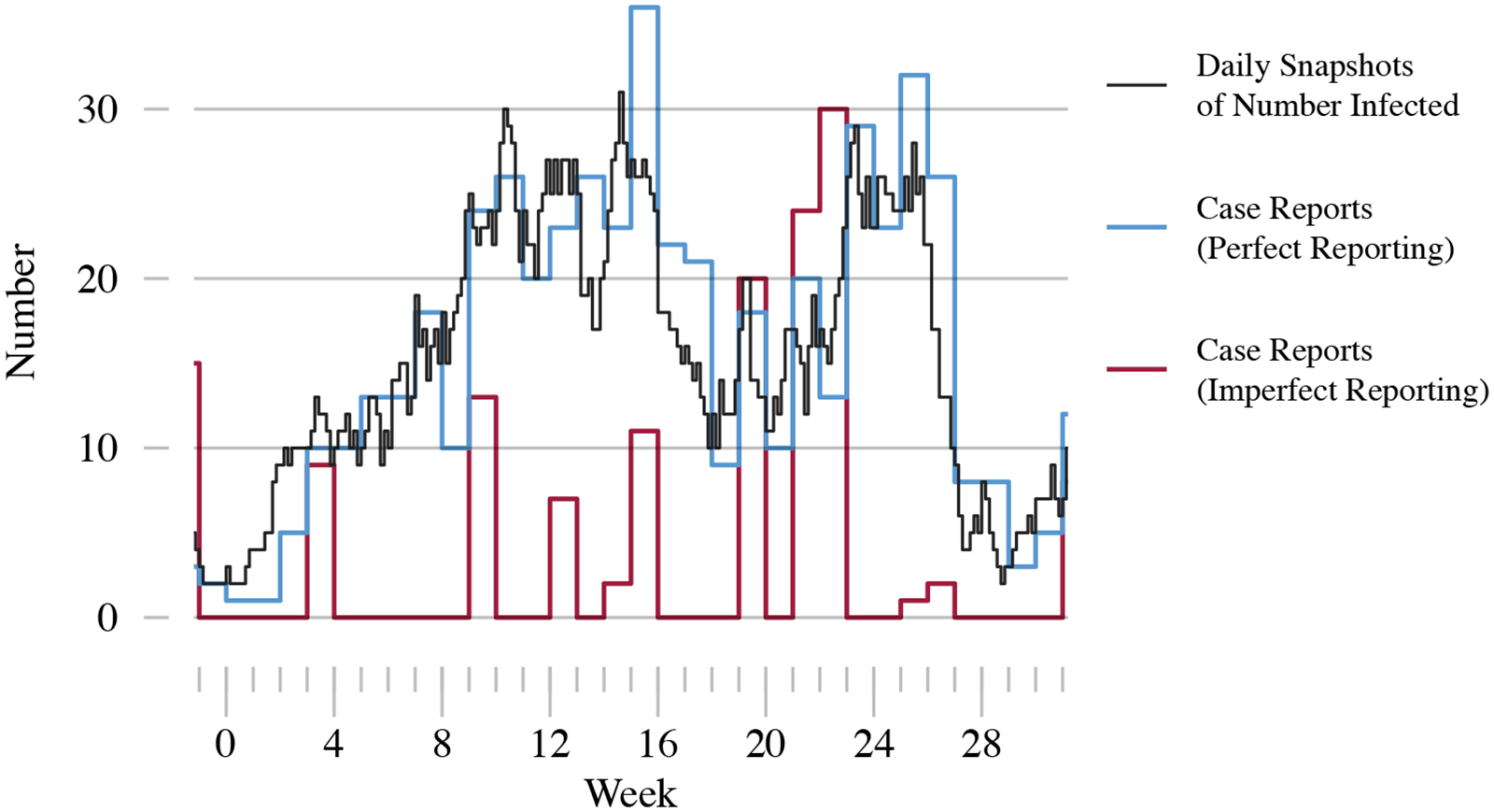
Example of aggregated data with reporting error. Three time series for the same simulated outbreak are shown: daily snapshots of the number of infected (black line), weekly aggregated case reports with no reporting error (perfect reporting, blue line) and weekly aggregated case reports with reporting error (imperfect reporting, red line). Reporting error is modeled using a negative binomial distribution with *ρ* = 0.25 and *ϕ* = 0.1. The high overdispersion (small *ϕ*) means that there is little visual correspondence between the number of case reports with and without reporting error. Additionally, the number of case reports can exceed the true number of cases (overreporting), in spite of the low reporting rate. SIR simulations performed using the same parameters as Fig 1.

**Fig 3:**
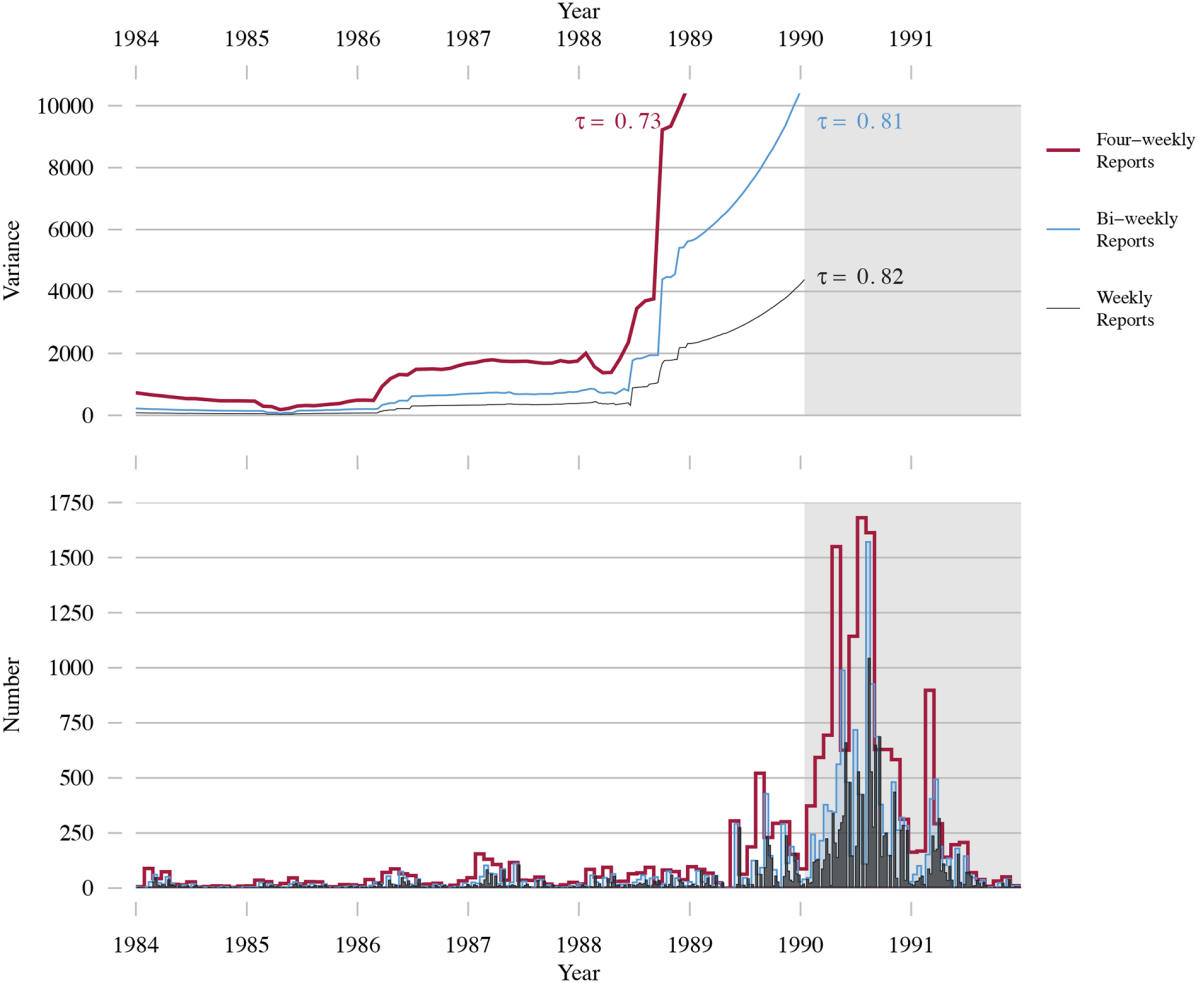
Lead up to an outbreak of measles in California. Weekly case report data from the US CDC’s MMWR compiled and released by Project Tycho [34]. Estimates for measles reporting efficacy in the USA are around 45% [27, 35, 36] In addition to the raw weekly case reports, the bottom row shows the data aggregated into bi-weekly and four-weekly reports. Changing the aggregation period changes not only the values of the variance (top row), but also the trend, quantified using Kendall’s *τ*. Only data to the left of the shaded area are included in calculating the variance. The moving average is calculated using a two year window, i.e bandwidth *b* = 52 for weekly reports; *b* = 26 for bi-weekly; *b* = 13 for four-weekly. For details see the Methods section.

A major obstacle to deploying early-warning systems is the type of data available to calculate the EWS. Theoretical predictions assume the data will be sequential recordings (or “snapshots”) of the true number of infected in the population through time [8–13]. In this paper we refer to this as snapshots data. However, epidemiological data originate instead from notifications by public health practitioners whenever a new case is identified. Public health bodies aggregate individual cases into regular case reports (e.g. the US Centers for Disease Control and Prevention’s Morbidity and Mortality Weekly Report), as shown in Fig 1. Different combinations of serial interval (difference in time of symptom onset between primary and secondary cases) and aggregation period lead to time series which have very different appearances. Even assuming perfect reporting, variability in both the incubation period and onset of clinical symptoms mean that snapshots data cannot be reconstructed from case report data. In addition to aggregation, case reports are subject to reporting error (see Fig 2). Underreporting may occur due to asymptomatic infection, poorly implemented notification protocols, or socio-political factors [25–29]. Misdiagnoses and clerical errors in the compilation of reports can result in both under- and over-reporting [30–32]. Due to self reporting and contact tracing, once an index case has been positively identified secondary cases are more likely to be diagnosed, which may lead to clustering in case reports. The combination of case aggregation and reporting error results in a mismatch between snapshots and imperfect epidemiological data. EWS, such as the variance (Fig 3, top panel), are affected by imperfect data (Fig 3, bottom panel) and may not display the characteristic trends that form the basis for detecting disease emergence. This provides reason to question the direct application of EWS to observed data.

In this paper we report on a simulation study aimed at investigating the robustness of a range of EWS to case report data. We simulated a stochastic SIR model of a pathogen emerging via increasing *R*_0_, and corrupted the simulated case reports by applying a negative binomial reporting error. The area under the curve (AUC) statistic was computed to quantify how well trends in an EWS identify emergence. We find that performance depends on both the EWS and the reporting model. Broadly, the mean, variance, index of dispersion and first differenced variance perform well. The autocorrelation, autocovariance and decay time perform well unless either i) the data are highly overdispersed or ii) the aggregation period is less than the infectious period. The coefficient of variation, kurtosis, and skewness have a more subtle dependence on the reporting model, and are not reliable. We conclude that seven of ten EWS perform well for most realistic reporting scenarios.

## Methods

### Simulating infectious disease transmission with imperfect data

The dynamics of disease spread in a host population are modeled as a stochastic process using an SIR model with birth and death [37]. The model compartments and parameters are listed in Table 1. Transition rates and effects are listed in Table 2. The basic reproductive number for the SIR model is *R*_0_(*t*) = *β*(*t*)/(*γ*+ *α*), where *β*(*t*) varies due to nondescript secular trends in the transmissibility. Simulated data are generated using the Gillespie algorithm [33], which simulates a sequence of transition events (infection, recovery, birth and death), and returns the number of individuals in each model compartment through time. The SIR simulations are of a population with average size *N*_0_ = 10^6^. The parameter *ζ* gives the rate at which new cases arise due to external sources, and is set to *ζ* = 1 per week. The death rate, *α*, is the reciprocal of the life expectancy, set to 70 years. Case counts, *C*_*t*_, are given by the number of recovery events (at rate *γI*_*t*_) within each aggregation period, and are included in the model as an additional variable (see Table 1).

**Table 1:** Model symbols.

Symbol	Definition
*S*	number of susceptible individuals
*I*	number of infected individuals
*R*	number of removed individuals
*C*	number of cases
*ζ*	importation rate
*β*(*t*)	transmission rate
*γ*	recovery rate
*α*	per capita birth and death rate
*N*_0_	average population size

**Table 2:** Transitions of the SIR process model. At the beginning of each aggregation period *C* is reset to 0.

Name	(Δ*S*, Δ*I*, Δ*R*, Δ*C*)	Propensity
birth of *S*	(−1, 0, 0, 0)	*αN*_0_
death of *S*	(−1, 0, 0, 0)	*αS*
death of *I*	(0, −1, 0, 0)	*αI*
death of *R*	(0, 0, −1, 0)	*αR*
importation	(−1, 1, 0, 0)	*ζS*/*N*_0_
transmission	(−1, 1, 0, 0)	*β*(*t*)*SI*/*N*_0_
recovery	(0, −1, 1, 1)	*γI*

Reporting error is applied to the case count at the end of each aggregation period by sampling a negative binomial distribution,

$$P\left(K_{t}=k|C_{t}\right)=\frac{\Gamma (\phi +k)}{k!\Gamma (\phi )}(\frac{\rho C_{t}}{\rho C_{t}+\phi }{)}^{k}(\frac{\phi }{\rho C_{t}+\phi }{)}^{\phi },$$ (1)

with reporting probability *ρ* and dispersion parameter *ϕ* [38]. Given *C*_*t*_ cases, the mean number reported is *μ*_*t*_ = *ρC*_*t*_. The variance is specified by the dispersion parameter via the relation $\sigma_{t}^{2}=\mu_{t}+\mu_{t}^{2}/\phi$. Increasing *ϕ* reduces the overdispersion of the data, so that for large *ϕ* the distribution of reports is approximately Poisson.

### Theoretically predicted EWS

Previous work has proposed a range of different EWS to anticipate dynamical transitions [8, 12–15, 17, 18]. The ten candidate EWS considered in this paper are listed in Table 3. We consider additional indicators to those most frequently studied in the EWS literature (the variance, autocorrelation and coefficient of variation). As *R*_0_ approaches 1, the mean number of cases caused by introductions rises, making it a potential EWS. The index of dispersion is a similar measure to the coefficient of variation, and is defined as the variance to mean ratio. The decay time (or correlation time) is a log-transform of the autocorrelation, which diverges as *R*_0_ approaches 1 (the definition of critical slowing down). In addition to the autocorrelation, which is normalized by the variance, we consider the unnormalized autocovariance. As both the autocorrelation and variance increase, the autocovariance may outperform these two measures. Theoretical results show the increase in variance accelerates as *R*_0_ approaches 1, suggesting the first differenced variance as a complementary EWS. Additionally we investigate the performance of two higher-order moments, the skewness and kurtosis.

**Table 3:** List of early-warning signals.

EWS	Mathematical definition	Estimator*	Theoretical prediction^†^	Correlation with *R*_0_
Mean	*μ*_*t*_ = *E*[*X*_*t*_]	${\hat{\mu }}_{t}=\sum_{s=t-(b-1)\delta }^{t+(b-1)\delta }\frac{X_{s}}{2b-1}$	$\frac{\zeta /\gamma }{1-R_{0}}$	+
Variance	$\sigma_{t}^{2}=E\left[{\left(X_{t}-\mu_{t}\right)}^{2}\right]$	${\hat{\sigma }}_{t}^{2}=\sum_{s=t-(b-1)\delta }^{t+(b-1)\delta }\;\;\;\frac{{\left(X_{s}-{\hat{\mu }}_{s}\right)}^{2}}{2b-1}$	$\frac{\zeta /\gamma }{{\left(1-R_{0}\right)}^{2}}$	+
Coefficient of variation	CoV_*t*_ = *σ*_*t*_/*μ*_*t*_	${\hat{\text{CoV}}}_{t}=\frac{{\hat{\sigma }}_{t}}{{\hat{\mu }}_{t}}$	(*ζ*/*γ*)^−1/2^	0
Index of dispersion	${\text{IoD}}_{t}=\sigma_{t}^{2}/\mu_{t}$	${\hat{\text{IoD}}}_{t}=\frac{{\hat{\sigma }}_{t}^{2}}{{\hat{\mu }}_{t}}$	$\frac{1}{1-R_{0}}$	+
Skewness	${\text{Skew}}_{t}=E\left[{\left(X_{t}-\mu_{t}\right)}^{3}\right]/\sigma_{t}^{3}$	${\hat{\text{Skew}}}_{t}=\frac{1}{{\hat{\sigma }}_{t}^{3}}\sum_{s=t-(b-1)\delta }^{t+(b-1)\delta }\;\;\;\frac{{\left(X_{s}-{\hat{\mu }}_{s}\right)}^{3}}{2b-1}$	(*ζ*/*γ*)^−1/2^(1+ *R*_0_)	+
Kurtosis	${\text{Kurt}}_{t}=E\left[{\left(X_{t}-\mu_{t}\right)}^{4}\right]/\sigma_{t}^{4}$	${\hat{\text{Kurt}}}_{t}=\frac{1}{{\hat{\sigma }}_{t}^{4}}\sum_{s=t-(b-1)\delta }^{t+(b-1)\delta }\;\;\;\frac{{\left(X_{s}-{\hat{\mu }}_{s}\right)}^{4}}{2b-1}$	(γ/ζ)(2 + *R*_0_)^2^ +3(1 − γ/ζ)	+
Autocovariance^‡^	ACov_*t*_(*δ*) = *E*[(*X*_*t*_ − *μ*_*t*_)(*X*_*t*−*δ*_ − *μ*_*t*−*δ*_)]	${\hat{\text{ACov}}}_{t}=\sum_{s=t-(b-1)\delta }^{t+(b-1)\delta }\;\;\;\frac{\left(X_{s}-{\hat{\mu }}_{s}\right)\left(X_{s-\delta }-{\hat{\mu }}_{s-\delta }\right)}{2b-1}$	$\frac{\zeta /\gamma }{{\left(1-R_{0}\right)}^{2}}e^{-(1-R_{0})\gamma \delta }$	+
Autocorrelation^‡^	${\text{AC}}_{t}\left(\delta \right)=\frac{{\text{ACov}}_{t}\left(\delta \right)}{\sigma_{t}\sigma_{t-\delta }}$	${\hat{\text{AC}}}_{t}=\frac{{\hat{\text{ACov}}}_{t}}{{\hat{\sigma }}_{t}{\hat{\sigma }}_{t-\delta }}$	*e*^−(1−*R*_0_)*γδ*^	+
Decay time^‡^	${\bar{\tau }}_{t}=-\delta /\text{ln}\left[{\text{AC}}_{t}\left(\delta \right)\right]$	${\hat{\bar{\tau }}}_{t}=-\delta /\text{ln}[{\hat{\text{AC}}}_{t}\left(\delta \right)]$	$\frac{1}{(1-R_{0})\gamma }$	+
First differenced variance^‡^	$\Delta \sigma_{t}^{2}=\sigma_{t}^{2}-\sigma_{t-\delta }^{2}$	${\hat{\Delta \sigma }}_{t}^{2}={\hat{\sigma }}_{t}^{2}-{\hat{\sigma }}_{t-\delta }^{2}$	$\frac{2\zeta /\gamma }{{\left(1-R_{0}\right)}^{3}}c\delta$	+

*For snapshots data *X*_*t*_ = *I*_*t*_, for case reports data *X*_*t*_ = *K*_*t*_.

^†^All BDI theory results calculated using the stationary approximation, assuming *R*_0_(*t*) = *ct* and *cδ* ≪ 1, see [13].

^‡^*δ* denotes one time step.

Functional expressions for the dependence of each EWS on *R*_0_ can be found using the Birth-Death-Immigration (BDI) process, a variation of the SIR model which neglects susceptible depletion (i.e. *S*_*t*_ = *N*_0_). The BDI process is a one-dimensional stochastic process, depending only on the number of infected *I*_*t*_, and possesses an exact mathematical solution (for full details see [13]). This allows expressions for the moments and correlation functions of *I*_*t*_ to be found (Table 3, fourth column). BDI theory predicts that most EWS (the mean, variance, index of dispersion, autocovariance, decay time and first differenced variance) are expected to grow hyperbolically as *R*_0_ approaches one. The autocorrelation is expected to grow exponentially, the kurtosis quadratically and the skewness linearly. The coefficient of variation is the only EWS which does not grow, instead remaining constant. We propose observing these trends in data as a basis for anticipating disease emergence. The numerical estimators used in this paper are listed in Table 3, third column, discussed in more depth below.

### Quantifying EWS performance

Theoretical predictions from the BDI process are based on *I*_*t*_ and do not take into account effects of reporting error and aggregation. The focus of this paper is to examine the robustness of each EWS to reporting process parameters, using simulated case report data, *K*_*t*_. BDI theory predicts that 9 out of 10 EWS increase as the transition is approached. We quantify the association of each EWS with time using Kendall’s rank correlation coefficient [19]. A coefficient close to (+/−)1 implies consistent increases/decreases of the EWS in time. As the underlying dynamics of the case reports are stochastic, the value of the rank correlation coefficient is itself a random variable. Multiple simulations of the test (emerging) and null (stationary/not emerging) scenarios result in two distributions of correlation coefficients for each EWS. We measure performance using the AUC statistic, defined as the overlap of the two distributions, and may be interpreted as the probability that a randomly chosen test coefficient is higher than a randomly chosen null coefficient, AUC = *P*(*τ*_test_ > *τ*_null_) [39, 40]. The name comes from one method of calculating it, the area under the receiver operating characteristic (ROC) curve, a parametric plot of the false positive rate against true positive rate as the decision threshold is varied [41].

Instead of explicitly calculating the ROC curve, the AUC can be efficiently calculated after ranking the combined set of test and null correlation coefficients by value [40],

$$\text{AUC}=\left[r_{\text{test}}-n_{\text{test}}\left(n_{\text{test}}+1\right)/2\right]/\left(n_{\text{test}}n_{\text{null}}\right),$$ (2)

where *r*_test_ is the sum of the ranks of test coefficients and *n*_test_ and *n*_null_ are the number of realizations of the test and null models respectively.

In this paper the AUC statistic quantifies how successfully an EWS distinguishes whether or not a disease is approaching an epidemic transition. An AUC = 0.5 implies that an observed rank coefficient value conveys no information about whether or not the disease is emerging, i.e. the EWS is ineffective. If the AUC < 0.5 then a decreasing trend in the EWS indicates emergence, whereas if AUC > 0.5 an increasing trend indicates emergence. A larger |AUC − 0.5| implies better performance, if |AUC − 0.5| = 0.5 the rank coefficient value classifies the two scenarios perfectly.

### Numerical estimators for EWS

The mathematical definitions of the EWS depend on expectations of the stochastic process, *E*[*f*(*X*)] (Table 3, second column). To calculate EWS from non-stationary time series data we use centered moving window averages with bandwidth *b* as estimators for expectation values. For example, the mean at time *t* is estimated using

$${\hat{\mu }}_{t}=\sum_{s=t-(b-1)\delta }^{t+(b-1)\delta }\frac{X_{s}}{2b-1},$$ (3)

where *δ* is the size of one time step. Near the ends of the time series (*t* < *bδ* and *t* > *T* − *bδ*), the normalization factor 2*b* − 1 is reduced to ensure it remains equal to the number of data points within the window. Applying Eq 3 to the time series for *X* results in a time series for $\hat{\mu }$. Certain EWS depend on others, for example the variance depends on the mean. EWS are therefore calculated iteratively, for example $\hat{\mu }$ is first calculated using Eq 3, and then ${\hat{\sigma }}^{2}$ is found using

$${\hat{\sigma }}_{t}^{2}=\sum_{s=t-(b-1)\delta }^{t+(b-1)\delta }\frac{{\left(X_{s}-{\hat{\mu }}_{s}\right)}^{2}}{2b-1}.$$ (4)

Estimators for each EWS are in Table 3. For snapshots data *X*_*t*_ = *I*_*t*_, and for case report data *X*_*t*_ = *K*_*t*_. Throughout this paper we use a bandwidth of *b* = 35 time steps (weeks or months depending on aggregation period). Results have been found to be similar for a bandwidth of *b* = 100 time steps.

### Experiment design

To quantify the sensitivity of each EWS to reporting process, we calculate the AUC from simulated data for a range of different model parameter combinations. The experimental design is fully factorial (i.e. considers all parameter value combinations). The following four parameters are varied: *(i)* the infectious period, 1/*γ*, which can be either 7 or 30 days, *(ii)* the reporting probability, *ρ* = 2^−8*x*^ for *x* in {0, 0.05, 0.1, …, 1}, *(iii)* the dispersion parameter, *ϕ*, which is one of {0.01, 1, 100}, *(iv)* the aggregation period, *δ*, which is either weekly or monthly.

For the test model, the disease emerges over *T* = 20 years, via an increase *R*_0_. For the null model, *R*_0_ is constant. One epidemiological interpretation for the test scenario is it models transmission in a population with high vaccine coverage, where gradual pathogen evolution results in increasing evasion of host immunity. An alternative interpretation is it models zoonotic spillover, where pathogen evolution within an animal reservoir results in gradually increasing human transmissibility [42]. In both interpretations, the null model assumes no change in host-to-host transmissibility.

The transmission dynamics were simulated using the Gillespie algorithm [33]. The Gillespie algorithm assumes all model parameters (including the transmissibility) are constant. To simulate disease emergence we modify the Gillespie algorithm, discretely increasing *β* at the end of each day and after each reaction to ensure an approximately linear increase in *R*_0_ over *T* = 20 years, from *R*_0_(0) = 0 to *R*_0_(*T*) = 1. For the null model, transmission is simulated for 20 years at a constant rate, *R*_0_ = 0. Our choice of null has no secondary transmission, making the classification problem easy under perfect reporting. This enables clearer identification of responses to reporting process effects as results span the full range of the AUC statistic. We repeated the experiment with null model *R*_0_ = 0.5, and found no qualitative differences.

For both scenarios transmission is subcritical, with disease presence maintained by reintroduction from an external reservoir. For each parameter combination 1000 replicates of both scenarios are generated.

We perform these computational experiments in *R* using the pomp package [43] to simulate the SIR model and the spaero package [44] to calculate the EWS. Code was written to simulate aggregation and reporting error. All code to reproduce the results is archived online at doi:10.5281/zenodo.1185284.

## Results

**Fig 4:**
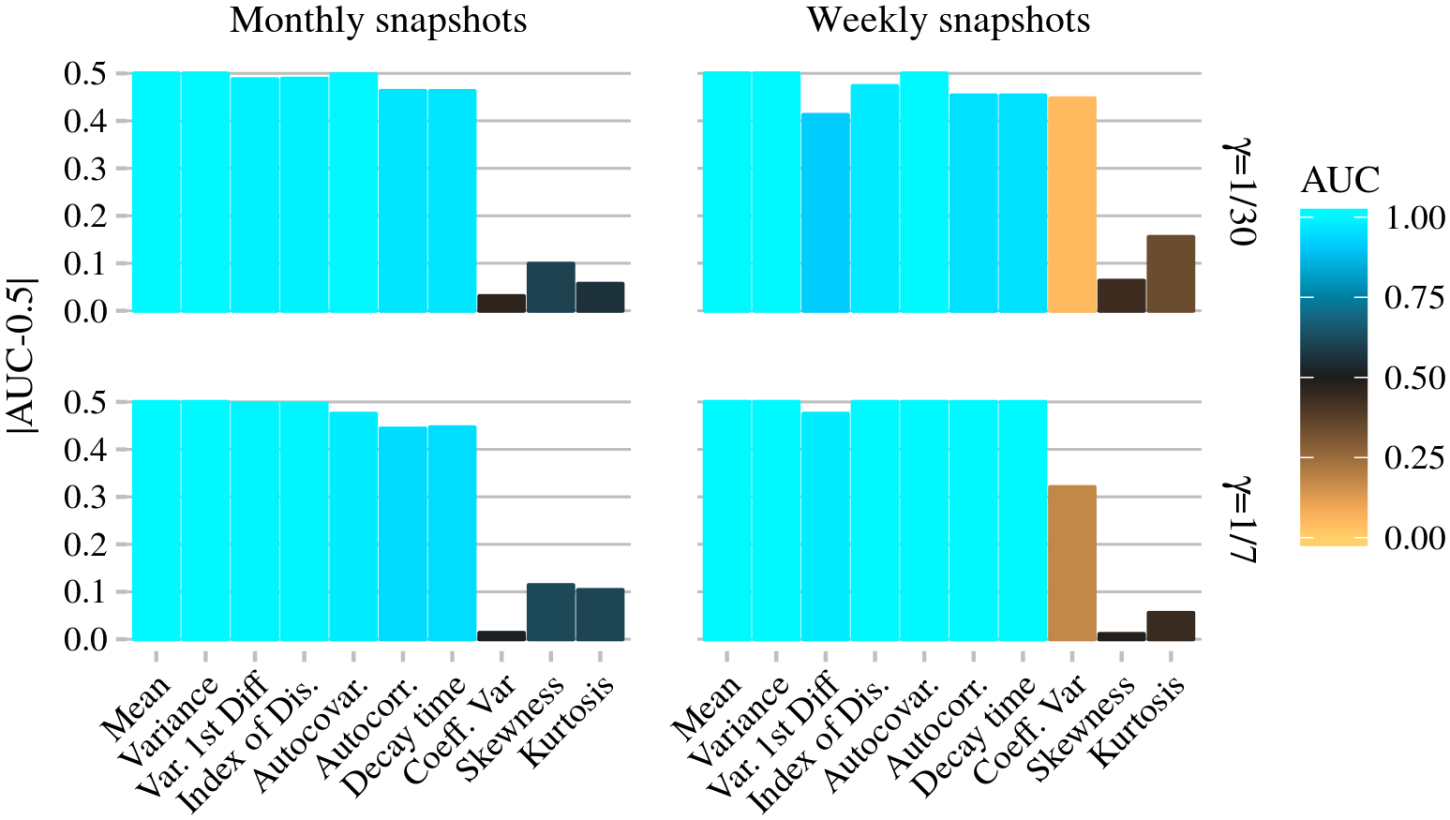
Performance of EWS calculated from weekly and monthly snapshots of the infectious population. AUC values further from 0.5 imply better performance. To investigate performance when the aggregation period is less than, equal to, and greater than the infectious period, results are shown for 1/*γ* = 1 week and 1 month. Simulations were performed using a stochastic non-fatal SIR model with birth and death. In a fully susceptible population, on average 1 susceptible individual per week acquires the infection from external sources. Individuals have a mean lifespan of 70 years. The average population size *N* = 10^6^ individuals. In simulations of the emerging scenario, *R*_0_ increases linearly from 0 to 1 over 20 years, in simulations of the stationary scenario *R*_0_ = 0. AUC values calculated using 1000 replicates of both models, see Methods.

**Fig 5:**
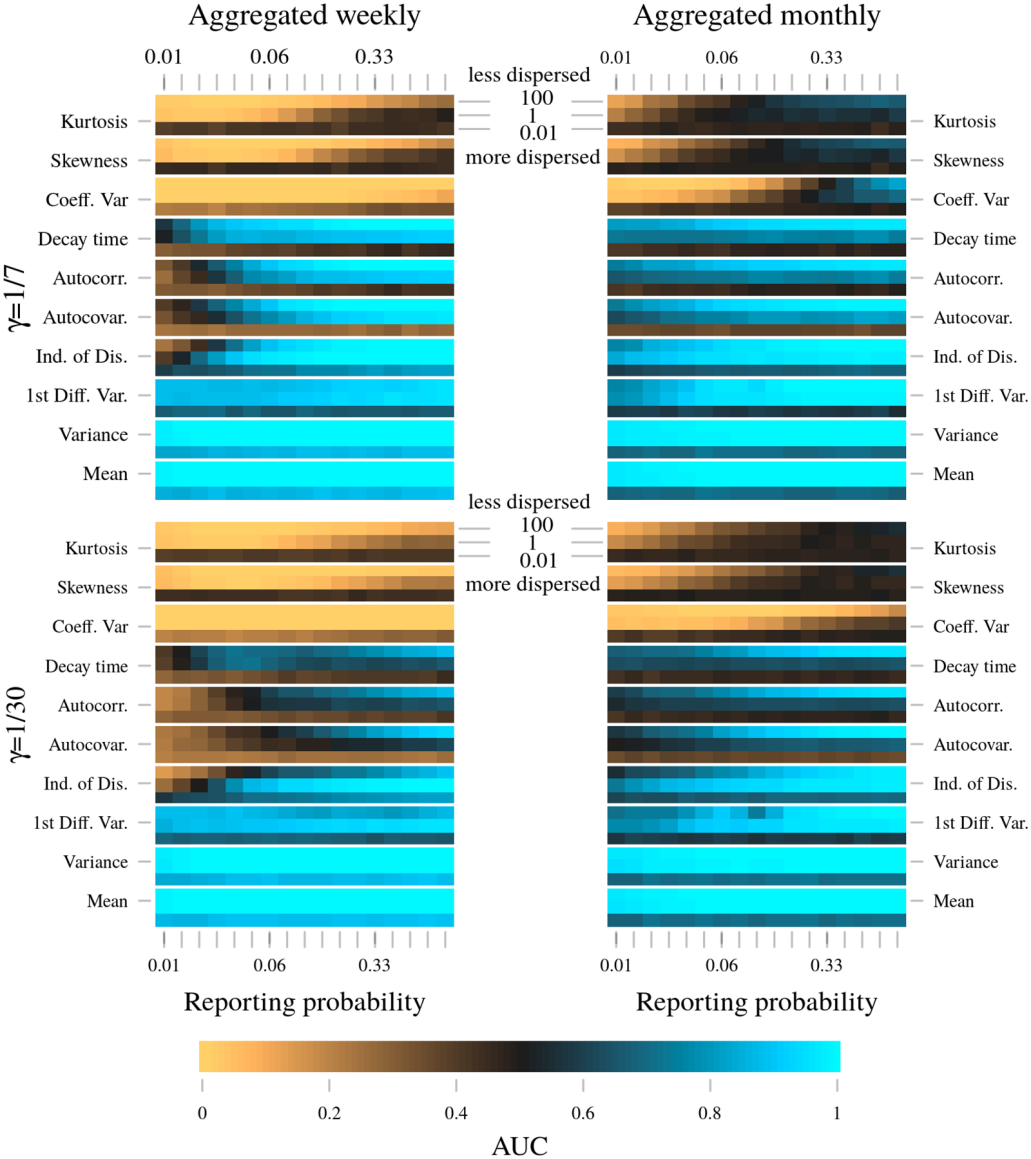
Heat maps showing impact of reporting process parameters and infectious period on performance of EWS. AUC values further from 0.5 imply better performance. For each reporting scenario, 1000 20 year long replicates of both the test (emerging) and null (stationary) SIR model are simulated using the Gillespie algorithm, for fixed model parameters see Methods. All EWS are then calculated for each replicate. To quantify ability to identify emergence, AUC values are calculated from the distributions of the rank correlation coefficient for each EWS, see Methods. The scales for both the overdispersion and reporting probability are logarithmic.

**Fig 6:**
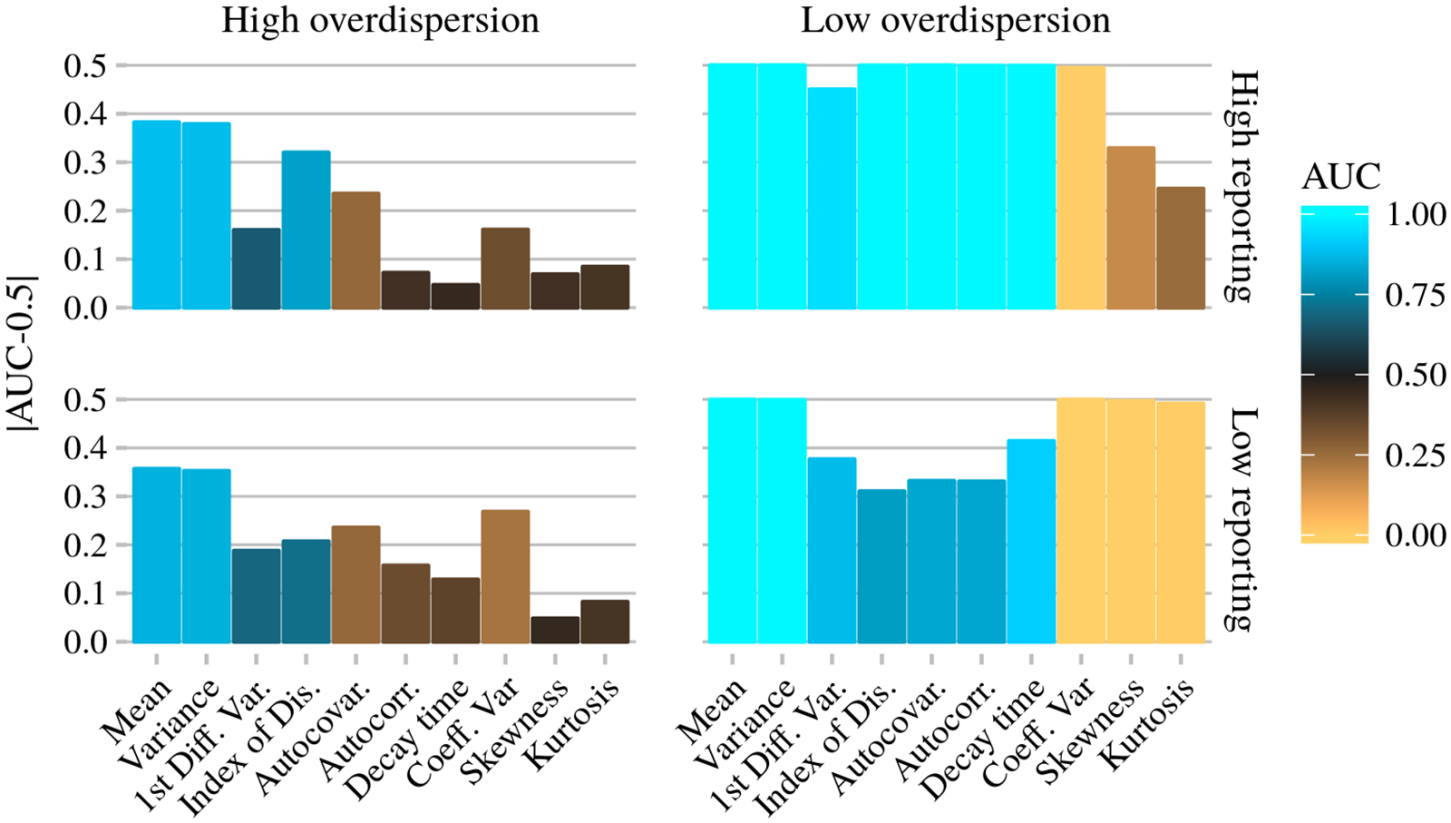
Performance of EWS under high/low reporting probability and high/low overdispersion scenarios. For high reporting *ρ* = 1.0 and low reporting *ρ* = 0.047. For high overdispersion *ϕ* = 0.01, for low overdispersion *ϕ* = 100. For all panels both the mean infectious period and aggregation period are 7 days. AUC values are from the same data set used in Fig 5.

AUC values are calculated for EWS using snapshots data (Fig 4) and case reports data (Figs 5 and 6). When calculated from snapshots data (the data type used in theoretical predictions), most EWS easily identify emergence (|AUC − 0.5| ≈ 0.5), with only small variation in performance with infectious period. The coefficient of variation, skewness and kurtosis are the exceptions. If the data are monthly snapshots (Fig 4, left column) they perform poorly (|AUC − 0.5| close to 0). If the data are weekly snapshots, then the skewness and kurtosis still perform poorly, however the coefficient of variation performs well. This improvement is particularly pronounced if the mean infectious period is 1 month (Fig 4, top right).

Fig 5 shows AUC values for each EWS when calculated using case report data. Each pixel in the heat maps corresponds to a distinct EWS and parameter combination. Based on the relationship between AUC value and reporting process parameters, we identify four groups of EWS.

Additional figures (S1–S4 Figs) show results for variations in experimental design. Qualitative findings were found to be robust to changes in: bandwidth *b*, initial *R*_0_ and emergence timescale *T*. Examples of the simulated time series are shown in S5 Fig, using the same reporting process parameters as in Fig 6.

### Coefficient of variation, skewness, kurtosis

Provided the data are aggregated monthly, with high reporting probability and low overdispersion, the coefficient of variation, skewness and kurtosis have similar AUC values when calculated from snapshots data (Fig 4) and case report data (Fig 5, right column). Unlike the other seven EWS, this it is not the case for weekly data. If calculated from weekly snapshots data with 1/*γ* = 1 week, the coefficient of variation has an AUC = 0.18 (Fig 4, bottom right). With reporting, if *ρ* = 1, *ϕ* = 100 the AUC = 0.005 (Fig 6, top right). By switching to case report data the performance of the coefficient of variation has improved dramatically. Similar improvements are seen for the skewness and kurtosis. In addition, and perhaps counterintuitively, these three EWS’s performances are further enhanced at lower reporting probabilities (compare the top right and bottom right panels of Fig 6). At low overdispersion and low reporting probability, the coefficient of variation (|AUC − 0.5| = 0.5) is joint with the mean and variance as the best performing statistic, closely followed by the skewness (|AUC − 0.5| = 0.497) and kurtosis (|AUC − 0.5| = 0.491).

The improvement in performance at low reporting probability is acutely sensitive to other model parameters. Both overdispersion in the reporting (for example Fig 6, left column) and larger aggregation period (Fig 5, right column) severely dampen the sensitivity to *ρ*. All three EWS perform poorly if *ϕ* = 0.01, regardless of the other model parameters.

### Autocovariance, autocorrelation, decay time

This group of EWS are all measures of the correlation between neighboring data points. At high reporting probability (*ρ* > 0.33) and low overdispersion (*ϕ* = 100), all three perform well (AUC > 0.77), regardless of infectious and aggregation periods (see Fig 5). Performance is comparable with snapshots data (Fig 4). Overall, they perform best if 1/*γ* = 1 week (Fig 5, top row) and worst if 1/*γ* > *δ* (Fig 5, bottom left). At low overdispersion, decreasing the reporting probability reduces the AUC (compare the top right and bottom right panels of Fig 6, AUC = 1.000 vs 0.831). The performance drop is largest if 1/*γ* = 1 month and *δ* = 1 week. The performance of all three EWS is negatively affected by overdispersion. Sensitivity to overdispersion is least for 1/*γ* = *δ* = 1 week, performance is only poor if *ϕ* = 0.01 and/or *ρ* ≲ 0.036 (Fig 5, top left). These three EWS are reliable indicators of emergence provided *δ* ≥ 1/*γ* and *ϕ* = 100.

### Mean, variance, first differenced variance

Unless reporting error is highly overdispersed (*ϕ* = 0.01), the mean, variance and first differenced variance perform extremely well (AUC ≈ 1, see Fig 5). If case reports are aggregated weekly and have high overdispersion (*ϕ* = 0.01), they are among the best performing EWS. The mean and variance have AUC > 0.85, and the first differenced variance has AUC ≈ 0.66, but is largely unaffected by reporting probability and infectious period. However, if case reports are aggregated monthly and *ϕ* = 0.01, then all three perform poorly. This holds regardless of reporting probability and infectious period, and is in line with the results for other EWS.

### Index of dispersion

The index of dispersion (unrelated to the dispersion parameter) has a similar performance to the previous group of EWS, however with certain differences. We first consider low overdispersion (*ϕ* = 100). At low reporting probabilities the index of dispersion performs best if 1/*γ* = 1 week and *δ* = 1 month (Fig 5, top right). For other combinations of infectious period and aggregation period, performance suffers a sharp drop as reporting probability decreases. This drop occurs at a reporting probability dependent on the infectious period and aggregation period, around *ρ* = 0.047 for *δ* = 1 week, and around *ρ* = 0.027 for *δ* = 1/*γ* = 1 month. Unique among the EWS, the index of dispersion performs best at intermediate overdispersion (*ϕ* = 1), in particular at small reporting probability. This is true for all infectious and aggregation periods, although most pronounced if 1/*γ* = 1 month and *δ* = 1 week. For *ϕ* = 0.01 the index of dispersion performs better if the data are aggregated weekly, and best if the infectious period is also one week, with AUC ≈ 0.71 for *ρ* = 0.047 (Fig 6, bottom left). Provided *ρ* ≳ 0.05 and *ϕ* > 0.01, performance is good for all aggregation and infectious periods. Overall performance is best if 1/*γ* = 1 week and *δ* = 1 month.

### Summary of results

Taken in isolation, the mean and variance are the EWS least impacted by reporting. Unless the overdispersion in the observation process is high (*ϕ* = 0.01), their performance is largely unaffected by reporting process parameters. At low reporting probabilities they outperform the autocorrelation, autocovariance, decay time and index of dispersion, and are independent of aggregation period and infectious period.

EWS sensitive to correlation between neighboring data points perform well unless i) *ϕ* = 0.01 and/or ii) 1/*γ* > *δ* and *ρ* ≲ 0.06. While it is clear how high overdispersion in reporting reduces correlation in the data, an explanation for ii) is less clear.

If calculated from snapshots data, the coefficient of variation, kurtosis and skewness are the worst performing statistics (|*AUC* − 0.5| ≈ 0). Using case report data improves performance under certain conditions. If cases are aggregated weekly with low reporting probability and low overdispersion then they are among the best performing EWS, with |*AUC* − 0.5| ≈ 0.5. In addition the trends of the skewness and kurtosis (both decreasing) are opposite those given by the BDI process (both increasing). Overall, we conclude that these three EWS are unreliable indicators of disease emergence as their performance is conditional on a limited range of reporting process parameters.

## Discussion

For mathematical reasons, proposed EWS for disease emergence have assumed access to regular recordings (“snapshots”) of the entire infectious population [8–13]. However, epidemiological data are typically aggregated into periodic case reports subject to reporting error. To examine the practical consequences of this mismatch between theory and data, in this paper we calculated EWS from case report data. We performed extensive numerical simulations to determine the sensitivity of each candidate EWS to imperfect data. Case aggregation and reporting error change the statistical properties of the data, and can have subtle effects on an EWS’s performance. We identified four groups of EWS based on their sensitivity to the various reporting process parameters. The performance of one group, consisting of the EWS with either polynomial or no growth with *R*_0_, has a nuanced relationship with the reporting process parameters. We therefore conclude that the coefficient of variation, kurtosis and skewness perform poorly as EWS. In general, the other EWS (the mean, variance, first differenced variance, index of dispersion, autocorrelation, autocovariance and decay time) all performed well and are strong candidates for incorporation in monitoring systems intended to provide early warning of disease emergence.

Surprisingly, the combination of reporting error and aggregation of data does not always have a detrimental effect on EWS performance. The coefficient of variation, kurtosis and skewness perform best when both reporting probability and overdispersion are low. At first glance this result appears counter-intuitive: as an increasingly large fraction of cases are missed, performance improves. The point to stress here is that by changing the parameters of the reporting process we are systematically changing the statistical properties of the time series. For instance, the BDI process predicts no trend in the coefficient of variation, due to the standard deviation and mean increasing with *R*_0_ at an identical rate [13]. With aggregation and reporting error this identity does not necessarily hold, introducing a trend in the coefficient of variation and improving its performance. To fully explain this phenomenon requires an analytical solution for the statistics of *K*_*t*_, which requires solving the stochastic process including aggregation and reporting error. However, it can be seen to be plausible if we focus only on stochasticity resulting from reporting error. For low overdispersion (e.g. *ϕ* = 100), the reporting probability distribution can be approximated by a Poisson distribution with parameter λ = *ρC*_*t*_. Ignoring demographic stochasticity, we replace *C*_*t*_ with *E*[*C*_*t*_] = *ηδ*(1 − *R*_0_)^−1^. Both the coefficient of variation and skewness for this distribution are λ^−1/2^ = {(1−*R*_0_)/*ρηδ*}^1/2^ and the kurtosis is λ^−1^ = (*ρηδ*)^−1^(1 − *R*_0_). These two expressions both decrease as *R*_0_ increases from 0 to 1, consistent with the experimentally observed AUC < 0.5. The improved performance at low *ρ* is a consequence of the increased stochasticity in reporting outweighing demographic stochasticity. Can these EWS be used to anticipate disease emergence? If overdispersion and reporting probability are known to be low, then yes. However, it is unlikely that the reporting process is sufficiently understood for an emerging disease. We conclude that these three EWS are unreliable and therefore not good indicators of emergence.

There is a similar reason for why the index of dispersion has a peak in performance at intermediate reporting overdispersion. The negative binomial reporting distribution, conditioned on *E*[*C*_*t*_] as above, has index of dispersion given by *σ*^2^/*μ* = 1 + *ρηδ*{*ϕ*(1−*R*_0_)}^−1^. Therefore increasing reporting overdispersion (i.e increasing *ϕ*^−1^) amplifies the response of *σ*^2^/*μ* to changes in *R*_0_. This leads to a greater differential, improving performance of the index of dispersion as an EWS. However, increased reporting overdispersion also implies increased volatility of data within a finite sized window, which reduces reliability. These two countervailing factors provide an explanation for the optimal performance at intermediate overdispersion values.

In our analysis we considered an SIR model with epidemiologically plausible parameters. The negative binomial distribution is meant to provide a stringent test on EWS performance, and the parameter ranges are conservative (especially for overdispersion). For instance, if there are 10 actual cases in a week, and reporting error is negative binomially distributed with *ρ* = 0.1 and *ϕ* = 0.01, then the mean number of reported cases is 1. However, the probability of no cases being reported is *P*(*K* = 0) = 0.955 whereas the variance in reported cases is *σ*^2^ = 101. The resulting time series is highly volatile, with little similarity in appearance to the underlying time series of actual cases. It is unlikely that case reports for an emerging disease will have such high overdispersion. In addition, for a highly pathogenic emerging disease, such as Middle East respiratory syndrome (MERS) or H7N9 avian influenza, the reporting probability is likely much higher than *ρ* = 1/256 (the smallest value we studied). Nonetheless, one of the encouraging findings of this study is that high reporting is not essential for reliable early warning. Clear trends in the EWS can still be identified, provided there are sufficiently many introductions for cases to be sporadically detected prior to emergence. These dynamics are typical for a reemerging vaccine controllable disease, such as measles, where cases are continually introduced into disease-free regions from endemic regions [45, 46].

Performance of EWS which depend on correlation between neighboring case reports was found to be contingent on the aggregation period being larger than the serial interval (equal to the infectious period for the SIR model). If this is not the case, there is a smaller probability that successive links in a chain of transmission fall into neighboring case reports. We speculate that this reduces the impact of fluctuations in a particular report on the subsequent report, diminishing their correlation. This effect is exacerbated if the reporting probability is low. A more rigorous explanation requires a full solution to the stochastic process with aggregation and reporting error. For many known pathogens the serial interval is larger than one week, for example measles virus and *Bordetella pertussis* [47]. For other pathogens it is less than one week, such as SARS coronavirus [48] and influenza virus [47, 49]. In order for the autocorrelation, autocovariance and decay time to be reliable EWS, our results suggest the data need to be aggregated by periods larger than the serial interval. The performance boost outweighs costs associated with having fewer data points. We expect that these three EWS will work best for pathogens with short serial intervals; for pathogens with extremely long serial intervals (such as HIV) reliable use of these EWS is unlikely.

The purpose of this study was not to identify the best EWS, but to investigate the robustness of this approach to the reporting process. In order to isolate the effects of incomplete reporting and aggregation error, we ignored parameter uncertainty by fixing epidemiological parameters (e.g. the infectious period and the introduction rate), rather than drawing them from a distribution. As shown in Table 3, both the mean and the variance scale with the introduction rate, which is a product of the *per capita* introduction rate and the susceptible population size. On the other hand, the index of dispersion, autocorrelation and decay time are all independent of introduction rate. Uncertainty of important factors, such as the susceptible population size, is a key challenge to anticipating emergence, and these three EWS may outperform the mean and the variance if uncertainty is included. Thus, while the mean and the variance are most robust to imperfect data, they are not necessarily the best EWS.

Instead, our results suggest that imperfect data is not a barrier to the use of EWS. One challenge to early-warning stems from the potential suddenness of novel pathogen emergence, for example SARS was unknown prior to the global outbreak in 2002-2003. For known pathogens, intermittent data availability presents a separate challenge. Mumps was excluded from the US CDC’s MMWR in 2002 following a period of low incidence. Subsequently, there was a series of large outbreaks, notably in 2006 in the Midwest, and mumps was reincluded. Methods such as EWS are contingent on surveillance efforts being maintained.

In addition to underlining the importance of disease surveillance, our work suggests ways it can be improved. Case reports sometimes include additional metadata, for example whether all suspected cases are counted or only clinically confirmed cases. The reporting error of case reports with differing case identification criteria is expected to be very different, as has been seen for instance with MERS [50]. This paper shows that EWS depend on the reporting process, and cross-validating EWS calculated from each data stream could improve performance. Provided it is available, how to leverage metadata is a promising avenue for future research into enhancing EWS.

These results provide an essential stepping stone from previous theoretically focused works to implementable early-warning systems. Our findings further reinforce the hypothesis that disease emergence is preceded by detectable EWS. While epidemiological factors preclude early-warning for certain pathogens, for example Ebola virus (estimates of *R*_0_ have consistently been greater than one [51]) and HIV (see above), they do not rule out many others, including reemerging childhood diseases [52], H7N9 avian influenza virus [53], and MERS coronavirus [54]. These pathogens all present public health risk, and EWS may be able to play an important role in monitoring for their emergence.

## Supporting information

S1 Fig Fig 5 repeated for *b* = 100. (TIFF)

S2 Fig Fig 5 repeated for 3 year bandwidth. (Weekly aggregation: ***b*** = **156**; monthly aggregation: ***b*** = **36**).
(TIFF)

S3 Fig Fig 5 repeated *R*_0_(0) = 0.5 for the test model and *R*_0_ = 0.5 for the null model. For both models, bandwidth ***b*** = **36**.
(TIFF)

S4 Fig Fig 5 repeated with *R*_0_(0) = 0.5 for the test model and *R*_0_ = 0.5 for the null model. For both models ***T*** = **10** years and bandwidth ***b*** = **36**.
(TIFF)

S5 Fig Example time series for the test model and null model. All parameters are the same as shown in Fig 6.
(TIFF)
