# Supplementary figures and images for: Anticipating epidemic transitions with imperfect data
Source: PLoS Comput Biol. 2018 Jun 8;14(6):e1006204. doi: 10.1371/journal.pcbi.1006204 (PMC6010299; doi:10.1371/journal.pcbi.1006204)

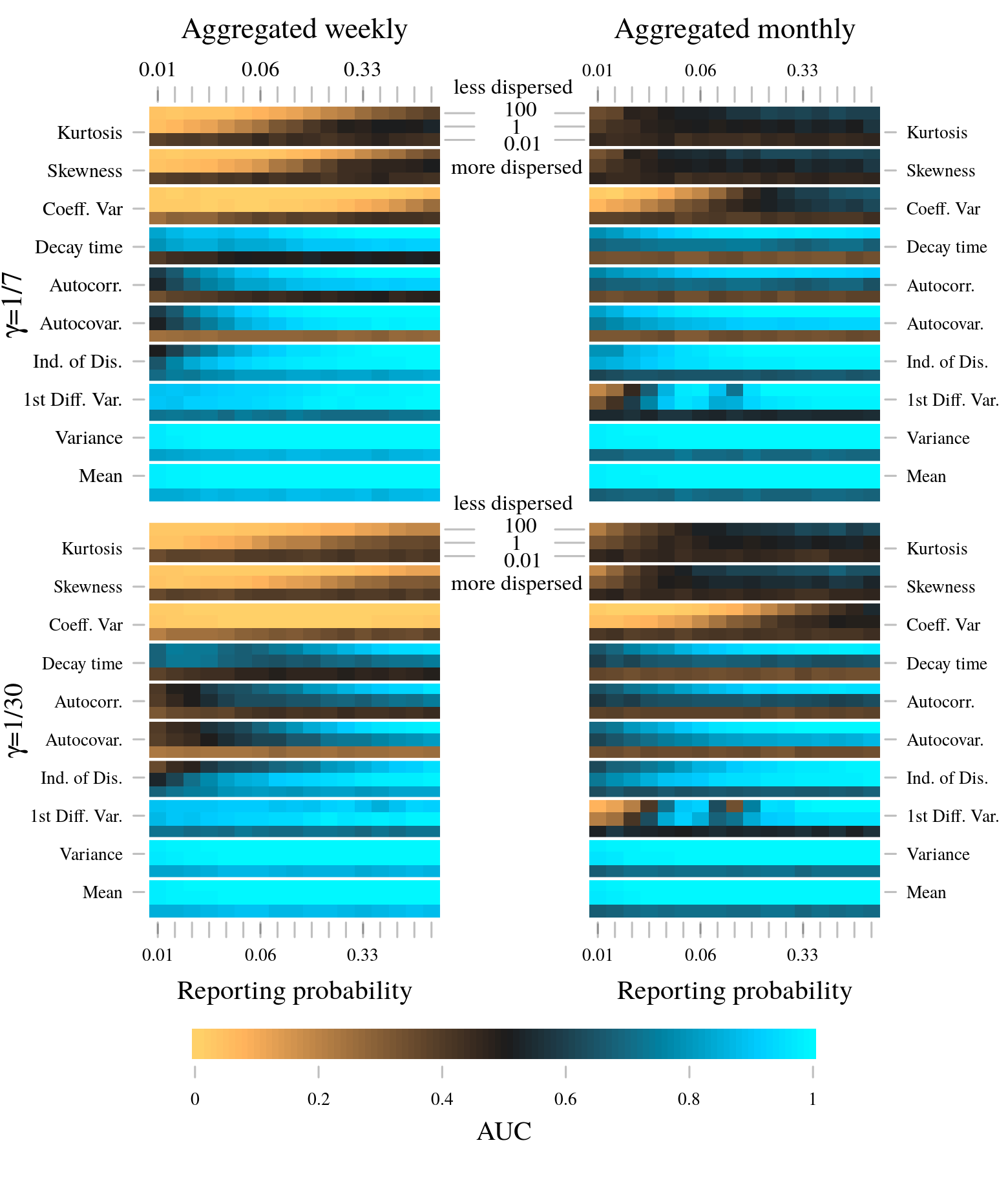

Supplement: S1 Fig — (TIFF) [file pcbi.1006204.s001.tiff]

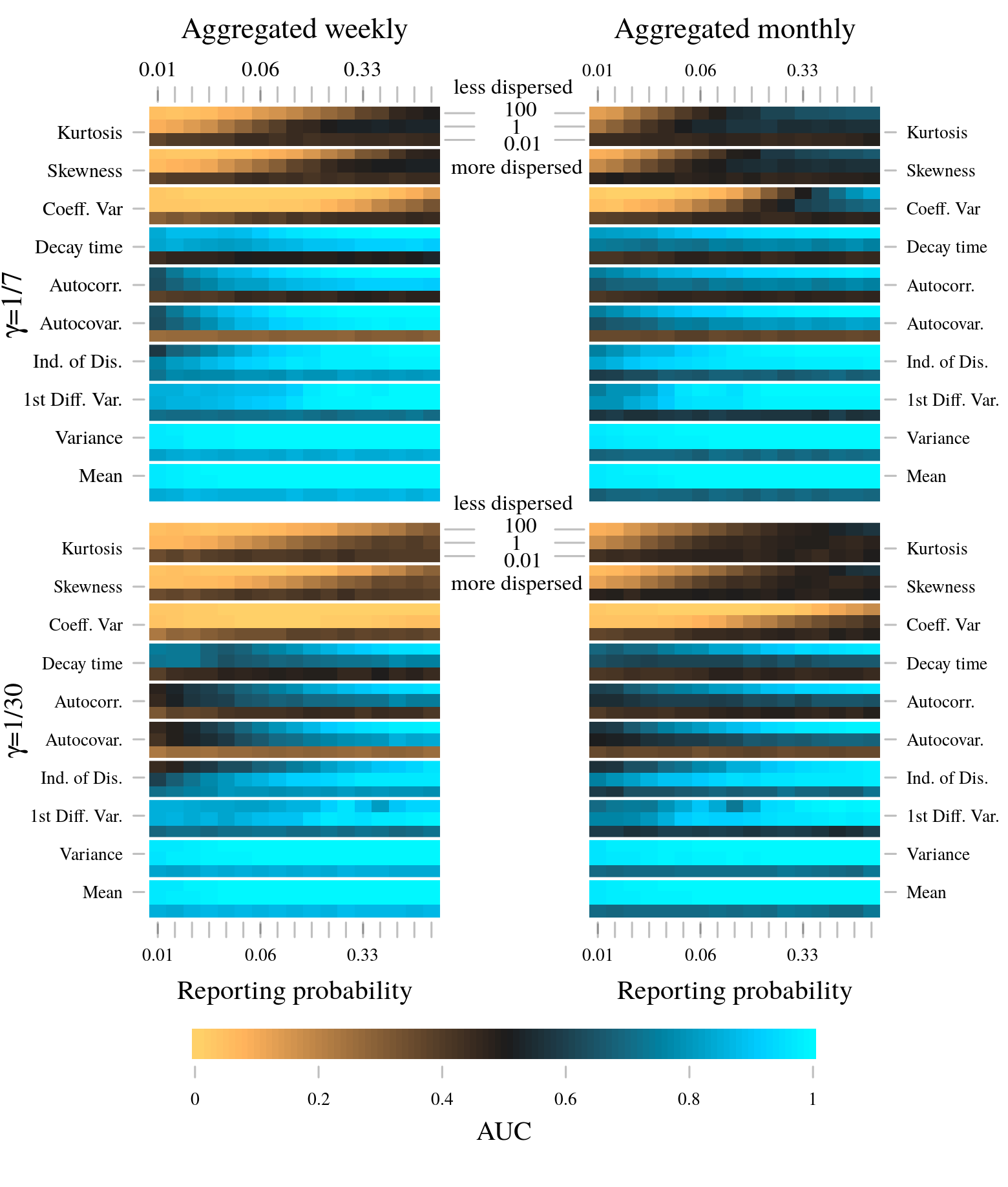

Supplement: S2 Fig — (Weekly aggregation: b = 156; monthly aggregation: b = 36). (TIFF) [file pcbi.1006204.s002.tiff]

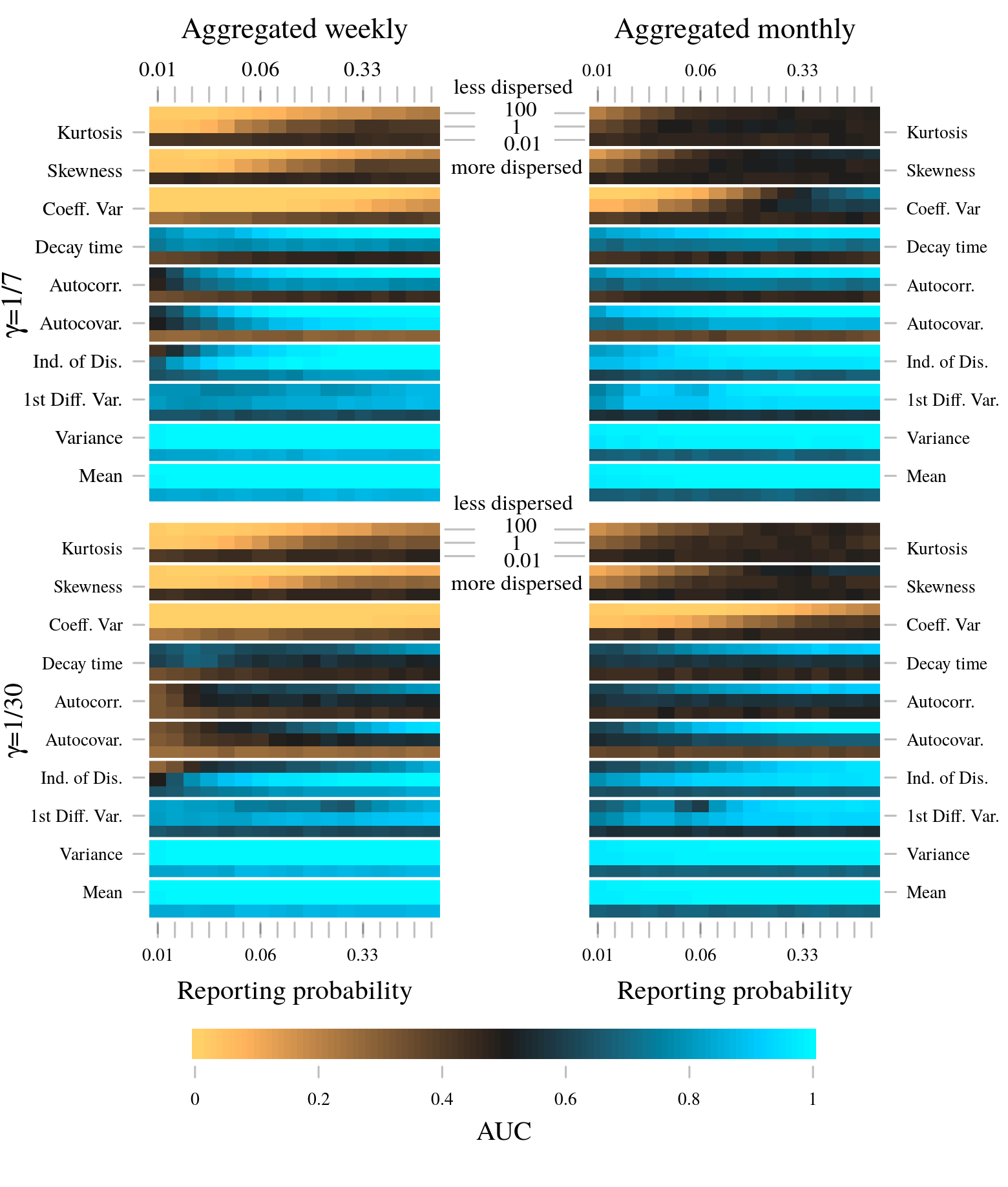

Supplement: S3 Fig — For both models, bandwidth b = 36. (TIFF) [file pcbi.1006204.s003.tiff]

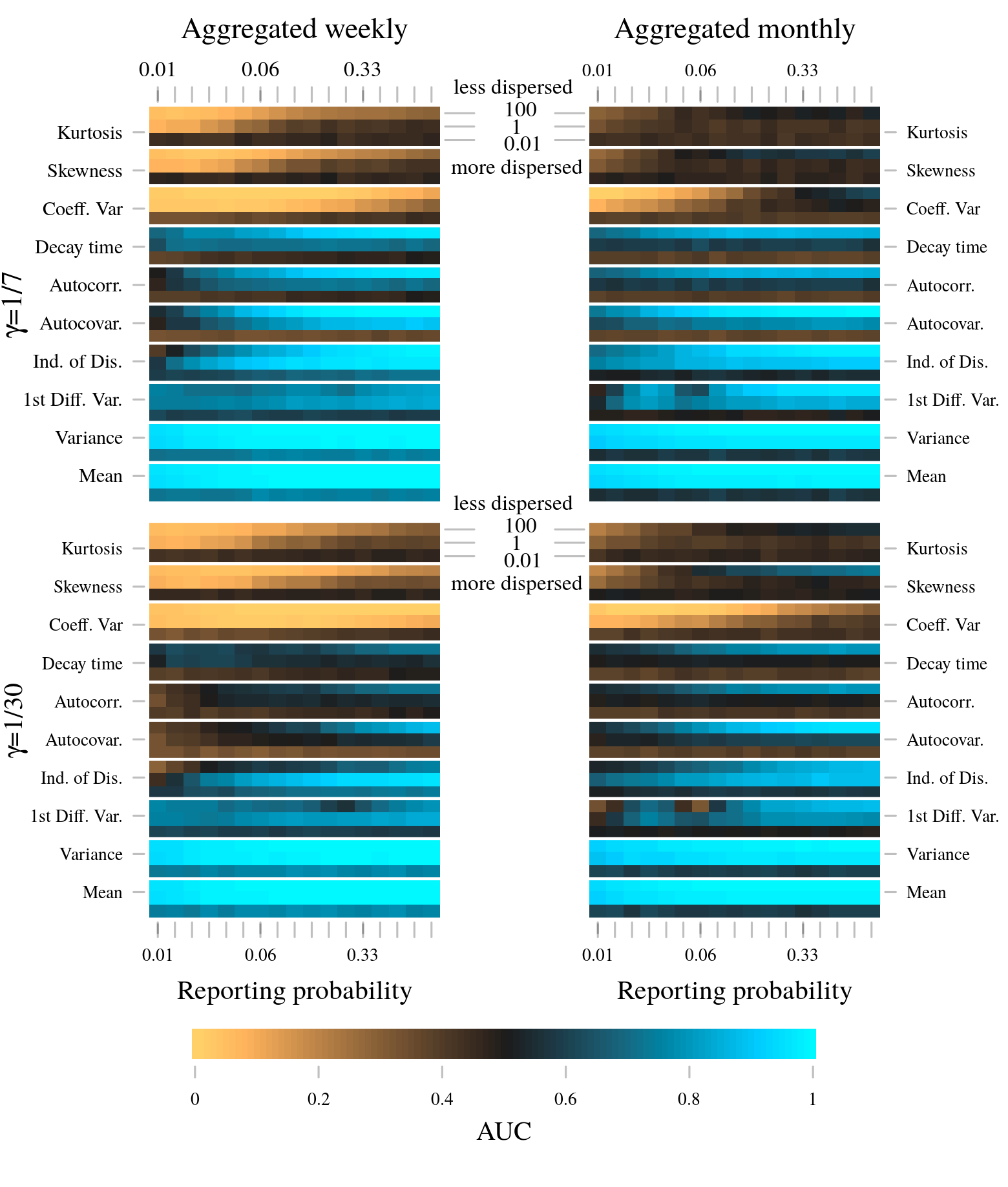

Supplement: S4 Fig — For both models T = 10 years and bandwidth b = 36. (TIFF) [file pcbi.1006204.s004.tiff]

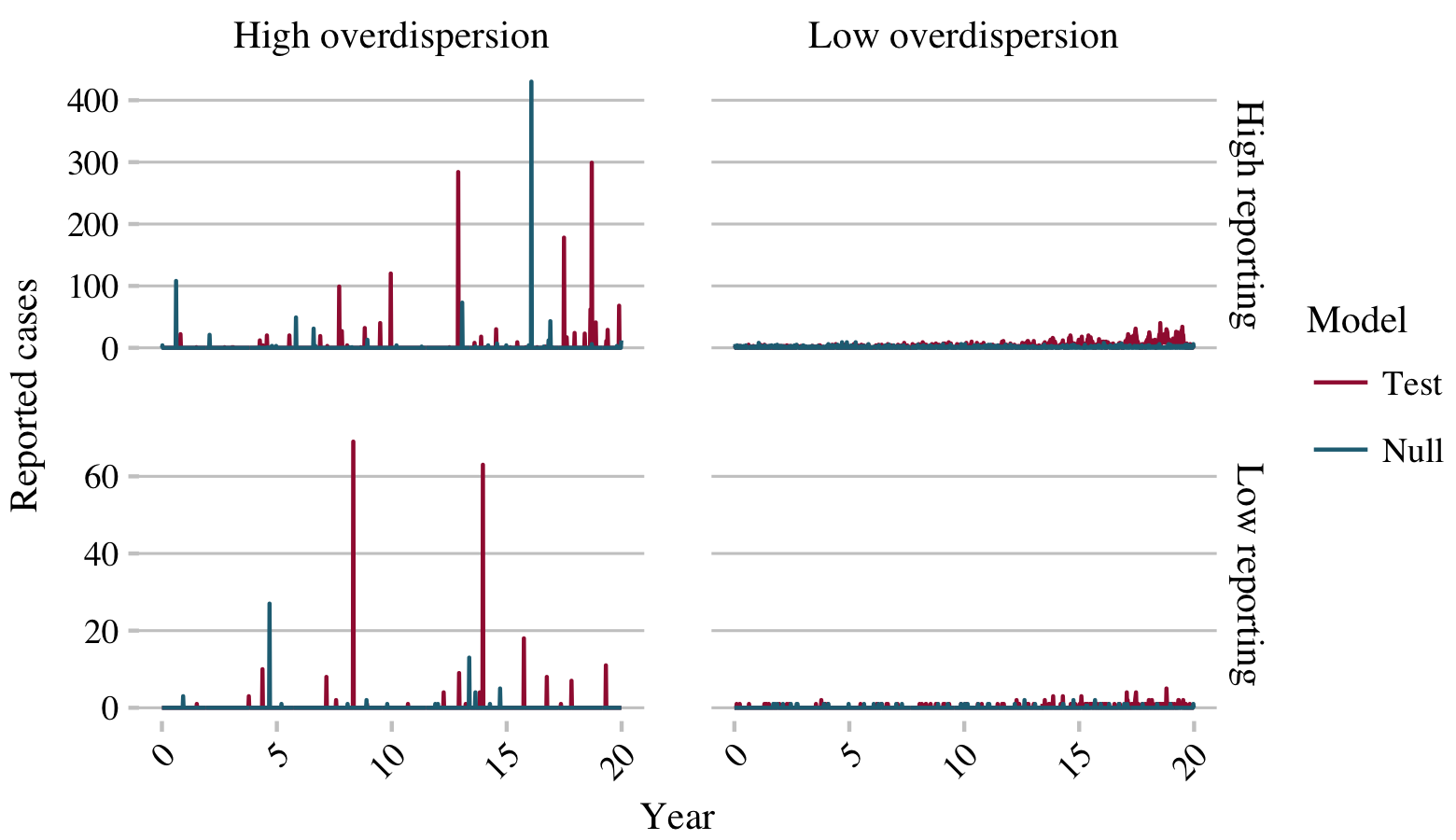

Supplement: S5 Fig — All parameters are the same as shown in Fig 6. (TIFF) [file pcbi.1006204.s005.tiff]
